# Deficient Multisensory Integration with concomitant resting-state connectivity in adult ADHD

**DOI:** 10.1192/j.eurpsy.2022.846

**Published:** 2022-09-01

**Authors:** M. Schulze, B. Aslan, S. Lux, A. Philipsen

**Affiliations:** 1 University Clinic Bonn, Dpeartment For Psychiatry And Psychotherapy, Bonn, Germany; 2 University of Bonn, Psychiatry, Bonn, Germany

**Keywords:** adult ADHD, Resting-state fMRT, multisensory Inegration

## Abstract

**Introduction:**

ADHD patients often report that they are being flooded by sensory impressions. Studies investigating sensory processing show hypersensitivity for sensory inputs across the senses. While studying unimodal signal-processing is relevant and well-suited in a controlled laboratory environment, our daily interaction with our environment does not occur merely unimodal. A complex interplay of the senses is necessary to form a unified percept. In order to achieve this, the unimodal sensory modalities are bound together in a process called multisensory integration (MI).

**Objectives:**

In the current study we investigate MI in an adult ADHD sample accompanied by resting-state functional magnetic resonance imaging (RS-fMRI).

**Methods:**

Twenty-five ADHD patients and twenty-four healthy controls were recruited. MI was examined using the McGurk effect, where - in case of successful MI - incongruent speech-like phonemes between visual and auditory modality are leading to a perception of a new phoneme. Mann-Whitney-U test was applied to assess statistical differences between groups. Resting-state functional MRI was acquired to realize a seed-to-voxel analysis

**Results:**

Susceptibility to MCGurk was significantly lowered for ADHD patients (ADHD_Mdn_:5.83%, Controls_Mdn_:44.2%, U= 160.5, p=0.022, r=-0.34). When ADHD patients integrated phonemes, reaction times were significantly longer (ADHD_Mdn_:1260ms, Controls_Mdn_:582ms, U=41.0, p<.000, r= -0.56). Seeded medio temporal gyrus was negatively associated in functional connectivity to primary auditory cortex, inferior frontal gyrus, precentral gyrus, and fusiform gyrus.

**Conclusions:**

MI seems to be deficient for ADHD patients for stimuli that need late attentional allocation. This finding is supported for higher functional connectivity from unimodal sensory areas to polymodal, MI convergence zones for complex stimuli.

**Disclosure:**

No significant relationships.

